# The Health of a Bacillus

**Published:** 1895-11

**Authors:** 


### SELECTIONS.

### The Health of a Bacillus.

Dr. C. H. Tebault, of New Orleans, in a recent edition of Gaillard's *Medical Journal*, quotes Professor Peter, of Paris, as follows: * * * "I have seen different germs produce indentical accidents. I can go further, and say, identical germs can produce different diseases. I have seen the bacterium coli, the virgule bacillus of Finkler, produce cholera. I have likewise seen the bacillus coli produce cholera, dysentery and typhoid fever. * * * From these facts I conclude that the bacillus is not unhealthy by itself, but that it may become so, and acquire new properties in the midst where it vegetates. * * * I have come to the conclusion that we ourselves, owing to some internal modifications, develop cholera and dysentery, and it is this change that modifies an innocent bacillus and endows it with toxic properties, that it may transmit to others. * * * The inoffensive bacillus is cholerized by the cholera."

In these days it behooves a healthy innocent bacillus to look a little out when he goes for his promenade lest he find his way into a dysenteric bowel and get poisoned. If this nefarious business of poisoning bacilli with the choleraic discharges and the diseased human excreta is not stopped the health of the bacilli as a body will be endangered and we will look to Professor Peter to suggest a method by which the continued innocence of bacillus may become an assured fact.—*Colorado Climatologist.*

